# Fish c-Jun N-Terminal Kinase (JNK) Pathway Is Involved in Bacterial MDP-Induced Intestinal Inflammation

**DOI:** 10.3389/fimmu.2020.00459

**Published:** 2020-03-30

**Authors:** Fufa Qu, Wenqian Xu, Zhangren Deng, Yifang Xie, Jianzhou Tang, Zhiguo Chen, Wenjie Luo, Ding Xiong, Dafang Zhao, Jiamei Fang, Zhigang Zhou, Zhen Liu

**Affiliations:** ^1^Hunan Provincial Key Laboratory of Nutrition and Quality Control of Aquatic Animals, Department of Biological and Environmental Engineering, Changsha University, Changsha, China; ^2^Key Laboratory for Feed Biotechnology of the Ministry of Agriculture, Feed Research Institute, Chinese Academy of Agricultural Sciences, Beijing, China

**Keywords:** c-Jun NH_2_-terminal kinases, AP-1 pathway, muramyl dipeptide, intestinal inflammation, *Ctenopharyngodon idella*

## Abstract

The c-Jun NH_2_-terminal kinases (JNKs) are an evolutionarily conserved family of serine/threonine protein kinases that play critical roles in the pathological process in species ranging from insects to mammals. However, the function of JNKs in bacteria-induced intestinal inflammation is still poorly understood. In this study, a fish JNK (*Ci*JNK) pathway was identified, and its potential roles in bacterial muramyl dipeptide (MDP)-induced intestinal inflammation were investigated in *Ctenopharyngodon idella*. The present *Ci*JNK was found to possess a conserved dual phosphorylation motif (TPY) in a serine/threonine protein kinase (S_TKc) domain and to contain several potential immune-related transcription factor binding sites, including nuclear factor kappa B (NF-κB), activating protein 1 (AP-1), and signal transducer and activator of downstream transcription 3 (STAT3), in its 5′ flanking regions. Quantitative real-time PCR results revealed that the mRNA levels of the JNK pathway genes in the intestine were significantly upregulated after challenge with a bacterial pathogen (*Aeromonas hydrophila*) and MDP in a time-dependent manner. Additionally, the JNK pathway was found to be involved in regulating the MDP-induced expression levels of inflammatory cytokines (IL-6, IL-8, and TNF-α) in the intestine of grass carp. Moreover, the nutritional dipeptide carnosine and Ala–Gln could effectively alleviate MDP-induced intestinal inflammation by regulating the intestinal expression of JNK pathway genes and inflammatory cytokines in grass carp. Finally, fluorescence microscopy and dual-reporter assays indicated that *Ci*JNK could associate with *Ci*MKK4 and *Ci*MKK7 involved in the regulation of the AP-1 signaling pathway. Overall, these results provide the first experimental demonstration that the JNK signaling pathway is involved in the intestinal immune response to MDP challenge in *C. idella*, which may provide new insight into the pathogenesis of inflammatory bowel disease.

## Introduction

Mitogen-activated protein kinases (MAPKs) are conserved serine/threonine protein kinases that are widespread across species, from yeasts to mammals, and transduce signals from outside the cell to the cytoplasm or nucleus to regulate stress response, inflammation, and other physiological processes (1–4). The MAPK signaling pathways are activated by sequential phosphorylation events through three-tiered cascades that consist of a MAPK, a MAPK kinase (MKK), and an MKK kinase (MKKK). The vertebrate MAPKs contain three main protein kinase subfamilies: extracellular signal-regulated kinases (ERKs), c-Jun NH_2_-terminal kinases (JNKs), and the p38 MAPK family (5, 6). Among them, JNKs, also known as stress-activated protein kinases (SAPKs), can respond to a wide variety of extracellular signal stimuli, including inflammatory cytokines, heat shock, and UV radiation (7–9).

JNKs form the most ancient and evolutionarily conserved family of MAPKs, and they play a vital role in the regulation of the stress response, cell apoptosis, and inflammatory responses (10–12). In vertebrates, the JNK family includes three members: JNK1 (MAPK8), JNK2 (MAPK9), and JNK3 (MAPK10) (13, 14); however, only one member has been reported in some invertebrate species, including shrimps (15), oysters (16, 17), and scallops (18). These JNK family members display different expression profiles: both *JNK1* and *JNK2* genes are broadly expressed in various tissues and cell types, while JNK3 mRNA is mainly detected in the brain and heart (19, 20). Similar to other MAPK members, JNKs possess the conserved function of dual phosphorylation of Thr and Tyr residues at their TPY motif of the activation loop. Upon stimulation, JNKs are activated by the phosphorylation of Thr and Tyr residues through specific MKKs (MKK4 or MKK7), which are upstream of the JNK pathway. Activation of JNKs results in the phosphorylation of transcription factors, such as activating protein 1 (AP-1), signal transducer and activator of downstream transcription 3 (STAT3), activating transcription factor 2 (ATF-2), and cAMP-response element binding protein (CREB), which are involved in regulating the transcriptional level of effector genes (21–25).

JNKs are multifunctional Ser/Thr protein kinases that have been reported to be involved in the modulation of numerous immunological responses to pathogenic infection in both vertebrates and invertebrates. Previous studies have revealed that the mammalian JNK cascade is activated by a variety of immune stimuli and is essential for the production of interleukins and interferons (26). In rat macrophages, it was demonstrated that JNK was involved in LPS- and *Escherichia coli*-induced IL-6 secretion via the MEK4-JNK-c-Jun signaling pathway (27). The antiviral activity of JNK was observed in the orange-spotted grouper (*Epinephelus coioides*), in which Ec-JNK3 inhibited SGIV infection and replication through the activation of ISRE and type I IFN reporter genes in the antiviral IFN signaling pathway (28). Additionally, JNK also can function as a positive regulator of the invertebrate immune system during an immune challenge. It was reported that *Drosophila* JNK participated in the immune deficiency (IMD) pathway, which is critical for the expression of downstream immune response genes during bacterial infection (29, 30). In mollusks, scallop JNK (*Py*JNK) was found to be involved in the immune defense response to Gram-positive bacteria (*Micrococcus luteus*) and Gram-negative bacteria (*Vibrio anguillarum*) infection (18). Recently, studies have demonstrated that oyster *Cg*JNK is activated by LPS stimulation and can regulate the transcription of the *Cg*IL17-1, *Cg*IL17-2, *Cg*IL17-4, and *Cg*IL17-6 genes in the hemocytes of *Crassostrea gigas* (17).

Intestinal inflammation frequently occurs due to extreme bacterial pathogen challenge conditions, which is a serious threat to intestinal health (31, 32). However, the exact pathogenesis of bacteria-induced intestinal inflammation is still not well understood in bony fish. Determining whether JNKs, as important components of the host immune defense system in species ranging from mammals to mollusks, play a potential role in intestine inflammation is worth further study. Therefore, the fish JNK (*Ci*JNK) pathway was identified, and its intestinal expression level in response to challenge with a bacterial pathogen (*Aeromonas hydrophila*) and muramyl dipeptide (MDP) was determined in *Ctenopharyngodon idella*. Additionally, the regulatory role of *Ci*JNK signaling in the MDP-induced expression levels of inflammatory cytokines in the intestine was also investigated. Moreover, the activation effect of *Ci*JNK on the AP-1 signaling pathway was analyzed in HEK293T cells. These data may contribute to a better understanding of the mechanisms of intestinal inflammation triggered by bacterial challenge.

## Materials and Methods

### Experimental Fish and Sample Collection

Healthy grass carps (weighing 30 ± 2 g) were collected from the Hunan Institute of Aquatic Science, Hunan Province, China, and cultured in a cage culture system at 25°C for a week before processing. Fish tissue samples, including the gill, intestine, kidney, blood, spleen, heart, muscle, and liver, were dissected for tissue distribution analysis. A series of developmental stage samples were collected from fertilized egg, gastrula, and neurula, as well as at organogenesis, hatching, and 1, 4, and 7 days post hatching (dph). The collected samples were frozen immediately in liquid nitrogen and then stored at −80°C until RNA isolation. All experiments were performed according to the recommendations of the Guidance of the Care and Use of Laboratory Animals in China. The research presented in this manuscript was approved by the Committee on the Ethics of Animal Experiments of Changsha University.

For the bacterial pathogen challenge experiments, healthy fish were randomly divided into three groups: one control group and two experimental groups. *A. hydrophila* (D-II-1) was kindly provided by the Feed Research Institute, Chinese Academy of Agricultural Sciences (33). The experimental individuals were intraperitoneally injected with 100 μl of live *A. hydrophila* (1.5 × 10^6^ CFU/ml) or MDP (10 μg/ml, Invitrogen); the control group was injected with 100 μl of PBS. Fish intestine from both the challenged and control groups was sampled at 0, 3, 6, 12, 24, 48, and 72 h post injection. The possible role of the JNK signaling pathway in MDP-induced intestinal inflammation was investigated by injecting grass carp with MDP (10 μg/ml, Invitrogen) for 24 h in the presence or absence of a JNK inhibitor SP600125 (100 μM, Sigma) for JNK. The intestines from three replicates were harvested for RNA extraction. To further investigate the regulatory function of the nutritional peptide on bacterial MDP-mediated intestinal inflammation, healthy fish were injected with PBS, MDP (10 μg/ml), MDP + carnosine (5 mM), or MDP + Ala–Gln (5 mM). After 24 h of treatment, the intestines from each group were harvested for gene expression level analysis.

### Total RNA Isolation and cDNA Synthesis

Total RNA was isolated from the harvested intestine and other fish tissues using RNAiso (Takara) according to the instruction manual. Genomic DNA was removed using DNase I (Sigma, USA). The integrity of isolated RNA was assessed by electrophoresis through a 1.2% agarose gel, and the purity was determined by measuring the absorbance at 260 and 280 nm using a BioPhotometer. PrimeScript™ 1st Strand cDNA Synthesis Kit (Takara, Japan) and PrimeScript™ RT Reagent Kit with gDNA Eraser (TaKaRa, Japan) were used to synthesize the cDNA template for gene cloning and expression analysis, respectively.

### Cloning and Sequencing of *Ci*JNK cDNA

A local BLAST search of the intestinal transcriptome data of grass carp showed one sequence that is homologous to the known JNK of *Megalobrama amblycephala* (MK315047.1) from the National Center for Biotechnology Information (NCBI). To obtain the open reading frame (ORF) sequence of *Ci*JNK, gene-specific primers, *Ci*JNK-F1 and *Ci*JNK-R1, were designed to amplify the cDNA sequence of *Ci*JNK using the identified sequence. The polymerase chain reaction (PCR) was performed on an Applied Biosystems™ Veriti 96-Well Thermal Cycler with a total volume of 50 μl containing 37.75 μl of ddH_2_O, 0.25 μl of TaKaRa Ex Taq DNA Polymerase (5 U/μl), 5 μl of 10× Ex PCR Buffer (Mg^2+^ Plus), 4 μl of dNTP mixture (2.5 mM each), 1 μl of each primer (10 μM), and 1 μl of cDNA template. The PCR temperature program was 94°C for 3 min followed by 35 cycles of 94°C for 30 s, 55°C for 30 s, and 72°C for 90 s, with a final extension at 72°C for 10 min. All the PCR products were analyzed by electrophoresis on 1.5% agarose gels and then cloned into the pMD19-T vector (TaKaRa, Japan). Positive bacterial clones were tested by colony PCR and sequenced with universal primers on an Applied Biosystems (ABI) DNA 3730 sequencer.

### Bioinformatic Analysis

The deduced amino acid sequence of *Ci*JNK was analyzed using the ORFfinder at NCBI (https://www.ncbi.nlm.nih.gov/orffinder/), and sequence identity/similarity analyses were performed with MatGAT v2.02. The isoelectric point (pI) and molecular weight (MW) were calculated by the Compute pI/Mw tool (http://web.expasy.org/compute_pi/). The functional sites and domains of the *Ci*JNK protein were predicted by the SMART program (http://smart.embl-heidelberg.de/). The three-dimensional (3-D) structure of the *Ci*JNK protein was modeled using the Swiss-Model software (https://swissmodel.expasy.org/). The potential transcriptional factor binding sites of the *Ci*JNK 5′-promoter region was analyzed by the AliBaba2 program (http://gene-regulation.com/pub/programs/alibaba2/index.html) and JASPAR server (http://jaspardev.genereg.net/). The exon–intron arrangement of *Ci*JNK was determined by using the Spidey tool (http://www.ncbi.nlm.nih.gov/spidey/). Multiple protein sequences were aligned using the MegAlign program via the Clustal W method in the DNASTAR software package. A phylogenetic tree was constructed using MEGA 5.05 using the neighbor-joining method with 1,000 bootstrap repetitions. The GenBank accession numbers corresponding to the JNK protein sequences examined are as follows: JNK (*C. idella*) AYN79349.1, JNK1 (*Homo sapiens*) AAI30571.1, JNK1 (*Rattus norvegicus*) NP_446281.2, JNK1 (*Bos mutus*) ELR52891.1, JNK1 (*Tyto alba*) KFV44724.1, JNK1 (*Apteryx rowi*) XP_025917082.1, JNK1 (*Danio rerio*) NP_571796.1, JNK1 (*E. coioides*) AIK19653.1, JNK1 (*Paralichthys olivaceus*) XP_019938631.1, JNK1 (*Perca flavescens*) XP_028423296.1, JNK2 (*H. sapiens*) AAH32539.1, JNK2 (*Mus musculus*) AAH28341.1, JNK2 (*Gallus gallus*) NP_990426.1, JNK2 (*Amazona aestiva*) KQK77114.1, JNK2 (*E. coioides*) ALK82291.1, JNK2 (*Takifugu rubripes*) XP_003970440.1, JNK2 (*Oryzias latipes*) XP_004073527.1, JNK2 (*D. rerio*) XP_001919688.1, JNK3 (*Pteropus vampyrus*) XP_011354691.1, JNK3 (*Bos taurus*) DAA28473.1, JNK3 (*Nestor notabilis*) KFQ50837.1, JNK3 (*Chaetura pelagica*) KFU83796.1, JNK3 (*Iconisemion striatum*) SBP22564.1, JNK3 (*Monopterus albus*) XP_020476746.1, and JNK3 (*D. rerio*) AAI09421.1.

### Quantitative Real-Time PCR Analysis

A relative quantitative real-time PCR (qRT-PCR) analysis was performed using a QuantStudio™ 3 Real-Time PCR System (Thermo Fisher, USA) to analyze the gene expression levels at different developmental stages, in various adult tissues, and during an immune challenge. The primer sequences used for the qRT-PCR assay are provided in Table 1. The real-time PCR assay was performed in a total volume of 16 μl that contained 5.72 μl of ddH_2_O, 0.64 μl of each primer (10 μM), 8 μl of 2× SYBR Premix Ex Taq II (Tli RNaseH Plus), and 1 μl of cDNA template. The qRT-PCR program was as follows: 95°C for 4 min, followed by 45 cycles of amplification at 94°C for 10 s, 55°C for 10 s, and 72°C for 10 s for signal collection in each cycle. After the PCR finished, dissociation curve analysis of the amplification products was performed to confirm that only one PCR product was present. The qRT-PCR data are expressed relative to the expression levels of the β-actin gene to normalize expression levels between the samples. All of the samples were analyzed in triplicate, and the expression values were calculated with the 2^−ΔΔCt^ method.

**Table 1:** Sequences of designed primers used in this study.

**Primer**	**Sequence (5′ to 3′)**	**Comment**
*Ci*JNK-F1	TCCTTTTATGAATCTGCTCTT	CDS Cloning
*Ci*JNK-R1	AAAAACTCTTACCTCCATTCT	
*Ci*JNK-F2	TCCTTTTATGAATCTGCTCTT	Real-Time PCR
*Ci*JNK-R2	TTTCTCACGCTTATTCCTGT	
*Ci*AP-1-F	AAAAGGATGTTCTGACTGGACT	Real-Time PCR
*Ci*AP-1-R	ATGTCCCCTGTTTTACTCCTAT	
*Ci*TNFα-F	CGCTGCTGTCTGCTTCAC	Real-Time PCR
*Ci*TNFα-R	CCTGGTCCTGGTTCACTC	
*Ci*IL-6-F	CAGCAGAATGGGGGAGTTATC	Real-Time PCR
*Ci*IL-6-R	CTCGCAGAGTCTTGACATCCTT	
*Ci*IL-8-F	ATGAGTCTTAGAGGTCTGGGT	Real-Time PCR
*Ci*IL-8-R	ACAGTGAGGGCTAGGAGGG	
*Ci*β-actin-F	GGCTGTGCTGTCCCTGTA	Real-Time PCR
*Ci*β-actin-R	GGGCATAACCCTCGTAGAT	
*Ci*JNK-F3	GATAAGAGCCCGGGCGGATCCATGAACAGGAATAAG	*Ci*JNK-Flag
*Ci*JNK-R3	ATCGAATTCCTGCAGAAGCTTTCACTGCTGCACCTG	
*Ci*MKK4-F1	GATAAGAGCCCGGGCGGATCCATGGCGACGTCCAGC	*Ci*MKK4-Flag
*Ci*MKK4-R1	ATCGAATTCCTGCAGAAGCTTTCAGTCCACGTACAT	
*Ci*MKK7-F1	GATAAGAGCCCGGGCGGATCCATGTCGTCGCTGGAG	*Ci*MKK7-Flag
*Ci*MKK7-R1	ATCGAATTCCTGCAGAAGCTTCTACCTGCTGAAGAG	
*Ci*JNK-F4	CTACCGGACTCAGATCTCGAGATGAACAGGAATAAG	*Ci*JNK-RFP
*Ci*JNK-R4	ATGGTGGCGACCGGTGGATCCCGCTGCTGCACCTGTG	
*Ci*MKK4-F2	CTACCGGACTCAGATCTCGAGATGGCGACGTCCAGC	*Ci*MKK4-GFP
*Ci*MKK4-R2	ATGGTGGCGACCGGTGGATCCCGGTCCACGTACATC	
*Ci*MKK7-F2	CTACCGGACTCAGATCTCGAGATGTCGTCGCTGGAG	*Ci*MKK7-GFP
*Ci*MKK7-R2	ATGGTGGCGACCGGTGGATCCCGCCTGCTGAAGAGA	
*Ci*MKK4-F3	GATCGCCAGGGATCCGTCGACTTATGGCGACGTCCAG	*Ci*MKK4-pACT
*Ci*MKK4-R3	GGTACCTGCGGCCGCTCTAGATCAGTCCACGTACAT	
*Ci*MKK7-F3	GATCGCCAGGGATCCGTCGACTTATGTCGTCGCTGGA	*Ci*MKK7-pACT
*Ci*MKK7-R3	GGTACCTGCGGCCGCTCTAGACTACCTGCTGAAGAG	
*Ci*JNK-F5	AATTCCCGGGGATCCGTCGACTTATGAACAGGAATAA	*Ci*JNK-pBIND
*Ci*JNK-R5	GGTACCTGCGGCCGCTCTAGATCACTGCTGCACCTG	

### Construction of Eukaryotic Expression Plasmids

The eukaryotic expression plasmids of *Ci*JNK, *Ci*MKK4, and *Ci*MKK7 were constructed and used for mammalian cell transfections. The ORF region of *Ci*JNK was cloned into pDsRed2-N1, pBIND, and pCMV-N-Flag using the ClonExpress® II One Step Cloning Kit (Vazyme, China) according to the manufacturer's protocol. The expression plasmids *Ci*MKK4-Flag, *Ci*MKK7-Flag, *Ci*MKK4-GFP, *Ci*MKK7-GFP, *Ci*MKK4-pACT, and *Ci*MKK7-pACT were also constructed for recombinant protein expression in HEK293T cells. The constructed recombinant plasmids were detected by restriction enzymes, colony PCR, and DNA sequencing. All the endo-free plasmids used for transfection were extracted by the HiPure Plasmid EF Mini Kit (Magen, China) according to the manufacturer's protocol.

### Cell Culture and Transient Transfection

HEK293T cells were used for subcellular localization and luciferase reporter analyses. Cells were cultured in DMEM (Gibco, USA) supplemented with 10% FBS (Gibco, USA), 100 IU/ml penicillin, and 100 mg/ml streptomycin (Gibco, USA) at 37°C under a humidified atmosphere of 5% CO_2_. The cultures were split every 2–3 days. For plasmid–liposome transfection, cells were seeded overnight and grown until they were 80–90% confluent at the time of transfection. The plasmids were transfected into cells in an Opti-MEM medium using Lipofectamine 2000 (Invitrogen) according to the manufacturer's instructions. After 6 h of transfection, the transfection mixture was replaced with a complete medium that consisted of DMEM containing 10% FBS.

### Subcellular Localization and Luciferase Reporter Assay

For the subcellular localization assay, plasmids with fluorescent tags—pEGFP-N1-*Ci*MKK4, pEGFP-N1-*Ci*MKK7, and pDsRed2-N1-*Ci*JNK—were transfected into HEK293T cells in a six-well cell culture plate. Forty-eight hours after transfection, the cells were washed twice with PBS buffer and then fixed with ice-cold paraformaldehyde (4%) at room temperature for 15 min. The nuclei were then stained with 1 μg/ml 4′,6-diamidino-2-phenylindole hydrochloride. Subsequently, the cells were washed with PBS again and observed using fluorescence microscopy (Leica Microsystems Heidelberg GmbH, Germany).

For the dual-luciferase reporter assays, HEK293T cells were co-transfected with the pRL-TK and AP-1-Luc reporter plasmids, as well as pCMV-N-Flag-*Ci*MKK4, pCMV-N-Flag-*Ci*MKK7, and pCMV-N-Flag-*Ci*JNK, to investigate the effect of *Ci*JNK on the transcriptional activity of AP-1. For the mammalian two-hybrid analysis, the expression vectors pBIND-*Ci*JNK, pACT-*Ci*MKK4, and pACT-*Ci*MKK7 were co-transfected with pG5-Luc in HEK293T cells to determine whether *Ci*JNK could directly interact with *Ci*MKK4 or *Ci*MKK7. All assays were performed with three independent transfections. At 48 h after transfection, Firefly and Renilla luciferase activities were measured using the Dual-Luciferase Reporter Assay System (Promega, USA) according to the manufacturer's instructions. Briefly, cells were washed twice with 200 μl of PBS and subsequently treated with 50 μl of 1× passive lysis buffer at room temperature for 15 min. Next, the Firefly and Renilla luciferase activities of cell lysates were measured by adding luciferase assay reagent II or 1× Stop & Glo reagent. Each experiment was performed in triplicate, and each assay was repeated at least three times.

### Statistical Analysis

All of the results are shown as the mean ± SD of different biological samples. The data were subjected to statistical evaluation with one-way analysis of variance (ANOVA), followed by the Tukey multiple comparison test using SPSS 17.0. Differences were considered statistically significant at *P* < 0.05 and extremely significant at *P* < 0.01.

## Results

### cDNA Cloning and Sequence Analysis of *Ci*JNK

**Figure 1:**
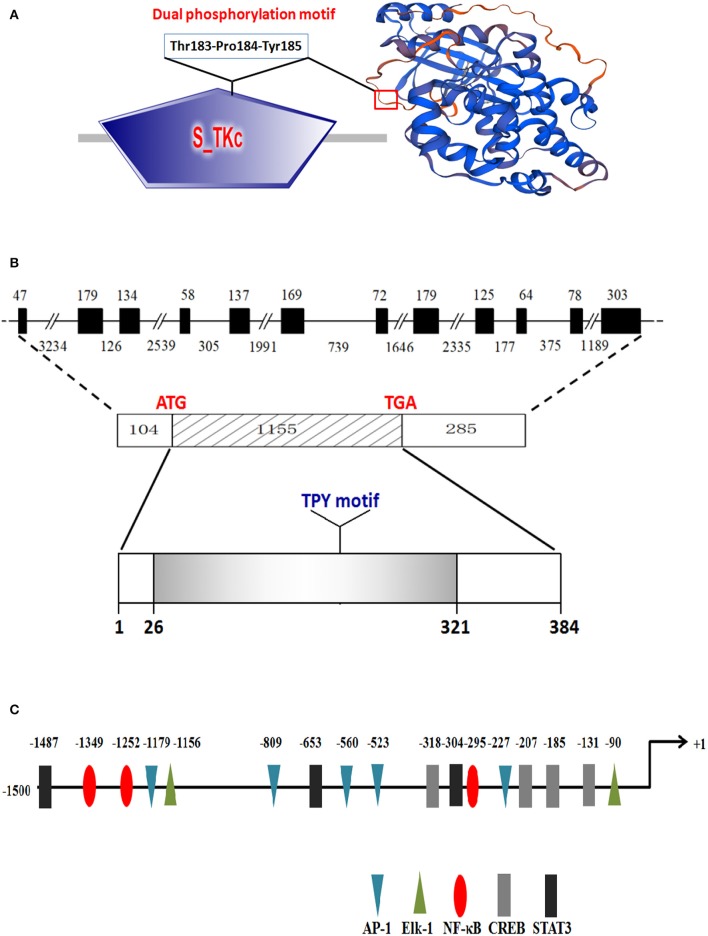
Protein structure and genomic organization of *Ci*JNK. **(A)** Domain organization and three-dimensional structure of *Ci*JNK as predicted by the SMART tool and Swiss-Model program. **(B)** Exons and introns are indicated by black boxes and black lines with the corresponding sizes (bp), respectively. **(C)** Identification of the putative transcription factor binding sites in the 5′-flanking regions (1,500 bp) of the *Ci*JNK gene. The positions of transcription factor binding sites are defined relative to the transcription start site (+1). The predicted transcription factor binding sites are shown by different colors of triangles, ellipses, or rectangles.

The cDNA sequence of *Ci*JNK was obtained by RT-PCR and submitted to the NCBI under GenBank accession number AYN79349.1. The cloned *Ci*JNK cDNA contains a 5′-UTR of 104 bp, a 3′-UTR of 286 bp, and an ORF of 1,155 bp that encodes a polypeptide of 384 amino acids with a theoretical pI of 6.84 and a calculated molecular mass of 44.19 kDa. A structural analysis based on the SMART tool and Swiss-Model program showed that the conserved domains and functional sites exist in the *Ci*JNK protein sequence, including a typical S_TKc domain (positions 26–321 aa) and a phosphorylation motif Thr–Pro–Tyr (positions 183–185 aa), with the typical features of JNK family proteins (Figure 1A). The genomic structure of *Ci*JNK was analyzed by comparing the genomic DNA and cDNA sequences. As shown in Figure 1B, the DNA sequence of *Ci*JNK possesses a multi-exonic gene structure containing 12 exons separated by 11 introns, and its mature mRNA sequence was generated by appropriate splicing. From the analysis of the 5′-upstream DNA sequence (~1.5 kb), several predicted transcription factor-binding sites were found in this region of the *Ci*JNK gene, including four CREB sites, three STAT3 sites, five AP-1 sites, three NF-κB sites, and two Elk-1 sites (Figure 1C).

**Figure 2:**
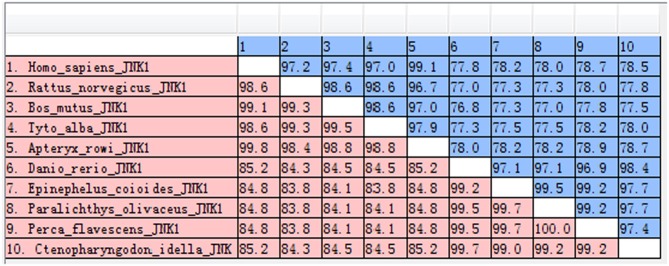
The similarities (red) and identities (blue) of JNK sequences were analyzed by the MatGAT v2.02 software.

**Figure 3:**
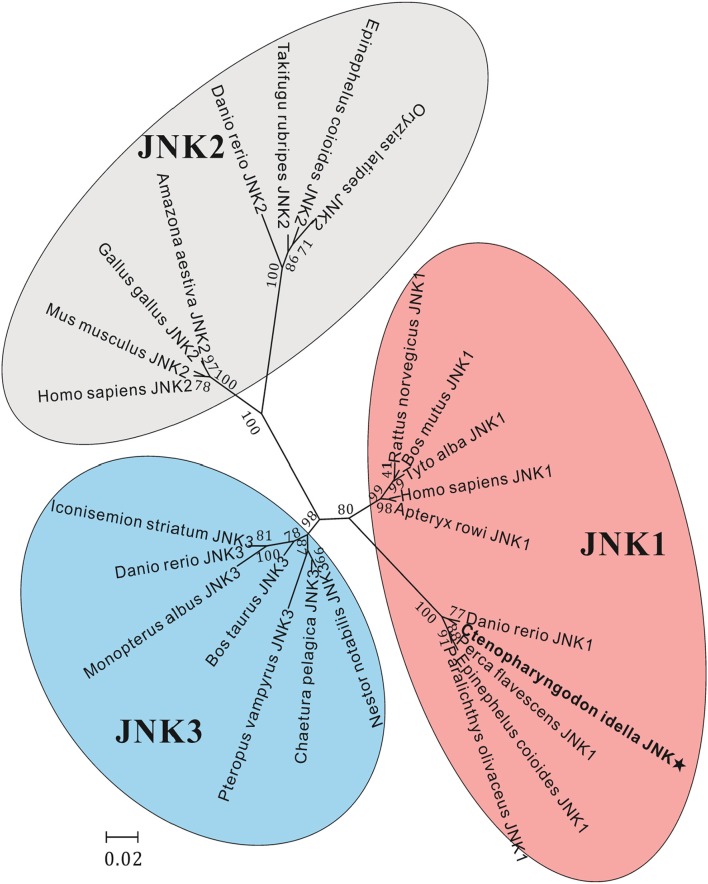
Phylogenetic analysis of JNK sequences, including JNK1, JNK2, and JNK3, was performed using the MEGA 5.05 software on the basis of the alignment of the complete amino acid sequences. The bar (0.2) represents the genetic distance. The numbers at each node refer to bootstrap values from 1,000 replications.

The amino acid sequence alignments further showed that highly conserved regions of *Ci*JNK and other JNKs exist in the S_TKc domain, and all of them contain a typical phosphorylation motif (Figure S1). A MatGAT analysis revealed that the deduced amino acid sequence of *Ci*JNK shares 84.3–99.7% similarity and 77.5–98.4% identity with JNKs from other species and that the highest similarity/identity was found to be with *D. rerio* (Figure 2). A phylogenetic tree classified the amino acid sequences of *Ci*JNK and other reported JNKs into three major groups (JNK1, JNK2, and JNK3), with the *Ci*JNK sequence located in the JNK1 group, suggesting that *Ci*JNK belongs to the JNK1 family (Figure 3). Moreover, phylogenetic analysis revealed that *Ci*JNK's closet evolutionary relationship is with JNK1 from *D. rerio*, consistent with the result of sequence alignment.

### Tissue- and Stage-Specific Expression Patterns of *Ci*JNK

**Figure 4:**
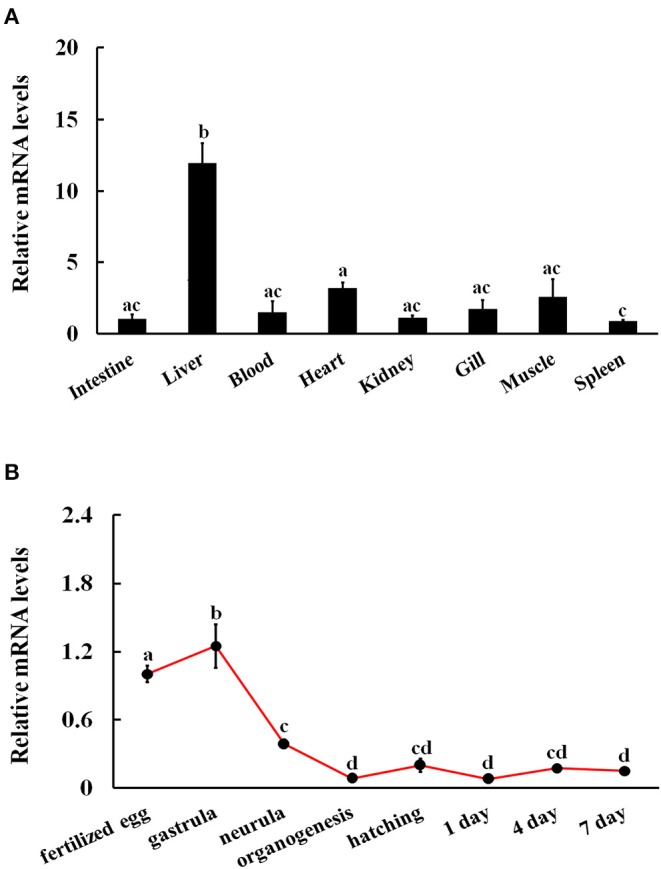
Relative expression levels of *Ci*JNK mRNA in different adult tissues **(A)** and developmental stages **(B)**. Grass carp β-actin was used to normalize mRNA levels in adult tissue and embryonic developmental stage samples. Each bar represents the mean of the normalized expression levels of the replicates (*N* = 3). Data without shared letters were significantly different (*P* < 0.05).

The mRNA expression of *Ci*JNK was detected via qRT-PCR for all examined adult tissues and developmental stages of grass carp. The qRT-PCR results revealed that *Ci*JNK was broadly expressed in the spleen, heart, gill, blood, intestine, kidney, muscle, and liver, with the highest expression levels in the liver and relatively low expression levels in the spleen, intestine, and kidney (Figure 4A). In addition, *Ci*JNK could be detected in all the selected samples from the embryonic stages of grass carp. Briefly, the level of *Ci*JNK mRNA significantly increased and peaked at the gastrula stage, then decreased significantly at the neurula and organogenesis stages, and finally maintained relatively low levels from hatching to 7 dph (Figure 4B).

### Expression of *Ci*JNK/*Ci*AP-1 Pathway Genes in Response to *A. hydrophila* Challenge

**Figure 5:**
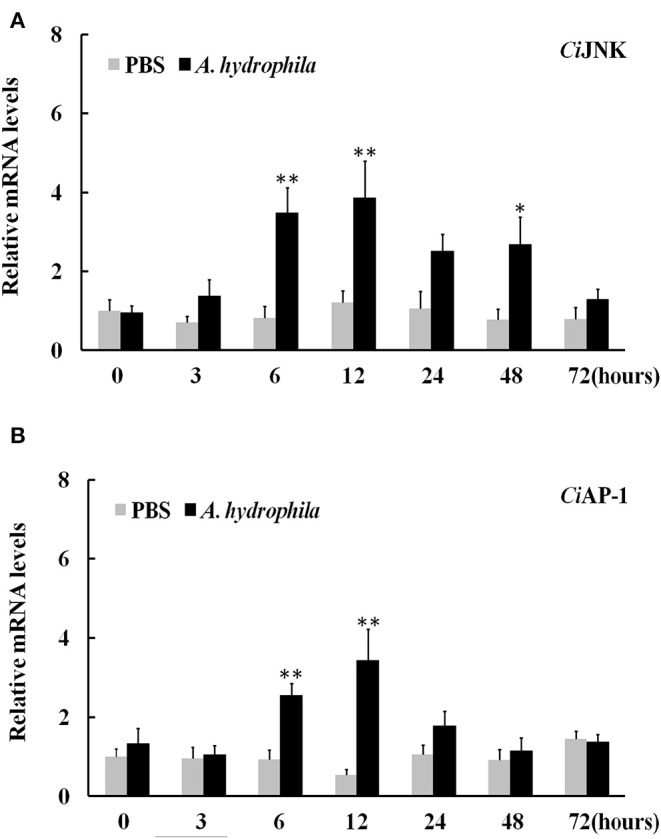
Relative expression of intestinal *Ci*JNK **(A)** and *Ci*AP-1 **(B)** in response to *Aeromonas hydrophila* challenge. The grass carp β-actin gene was used as a reference gene to normalize the expression level. Comparative analysis and statistical tests were performed on the challenge groups and PBS group at the same time point. Each bar represents the mean of the normalized expression levels of replicates (*N* = 3); significant differences are indicated by an asterisk (* and ** represent *P* < 0.05 and *P* < 0.01, respectively).

The expression profiles of JNK pathway genes in response to bacterial challenges were investigated by qRT-PCR analysis to measure the mRNA levels of intestinal *Ci*JNK and *Ci*AP-1 in response to *A. hydrophila* infection. The results showed that both *Ci*JNK and *Ci*AP-1 exhibited a strong and broad response to *A. hydrophila* challenge and that their expression levels were significantly upregulated in a time-dependent manner in the intestine of grass carp (Figure 5). Upon challenge with *A. hydrophila*, the expression level of intestinal *Ci*JNK initially significantly increased at 6 h post challenge (4.2-fold, *P* < 0.01) and peaked at 12 h post infection (5.4-fold, *P* < 0.01) compared with treatment with PBS. The mRNA level of *Ci*AP-1 in the intestine did not significantly increase until 6 h post challenge (2.7-fold, *P* < 0.01), reaching the highest level at 12 h post challenge (6.4-fold, *P* < 0.01). These data suggest that the grass carp JNK/AP-1 pathway may play an important role in the intestinal immune response to bacterial pathogen challenge.

### Expression of *Ci*JNK/*Ci*AP-1 and Inflammation Cytokines in Response to MDP Challenge

**Figure 6:**
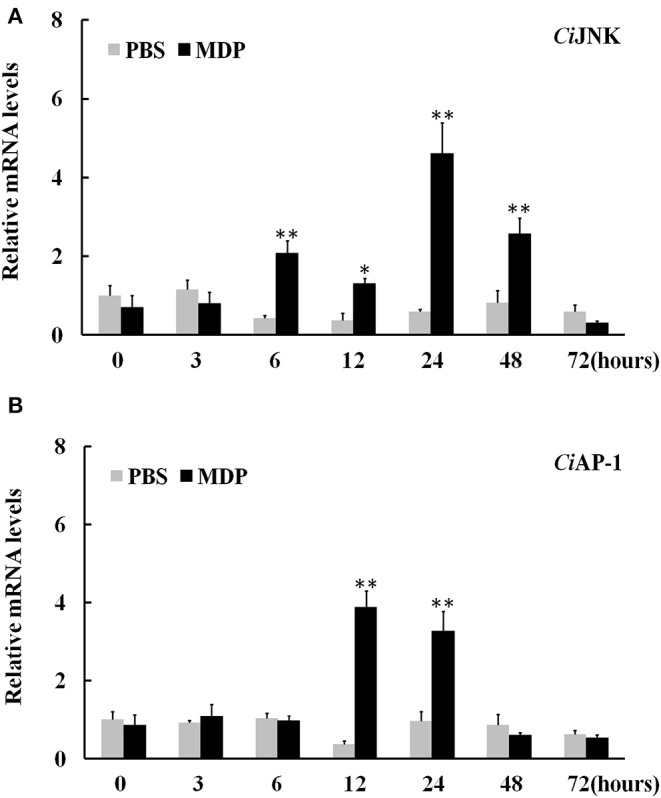
Relative expression of intestinal *Ci*JNK **(A)** and *Ci*AP-1 **(B)** in response to MDP challenge. The grass carp β-actin gene was used as a reference gene to normalize the expression level. Comparative analysis and statistical tests were performed on the challenge groups and PBS group at the same time point. Each bar represents the mean of the normalized expression levels of replicates (*N* = 3); significant differences are indicated by an asterisk (* and ** represent *P* < 0.05 and *P* < 0.01, respectively).

**Figure 7:**
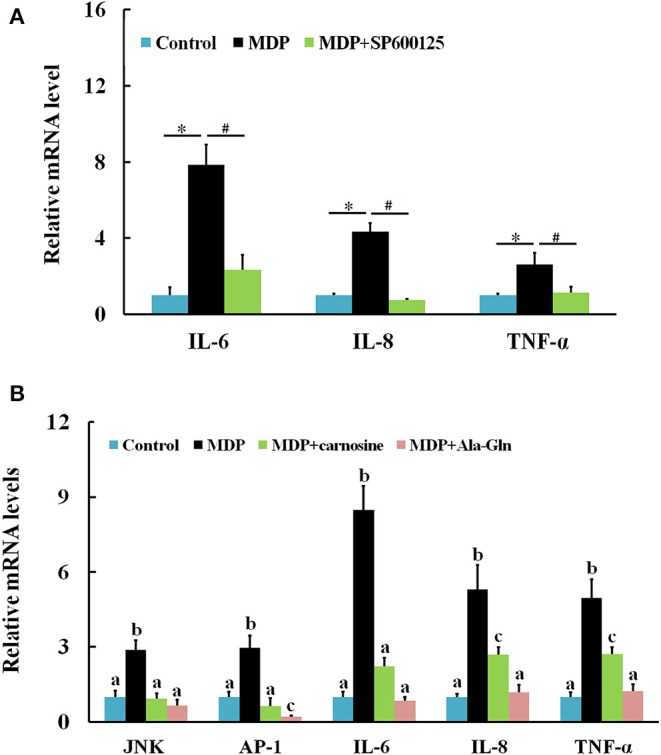
Relative expression of intestinal inflammatory cytokines and *Ci*JNK/*Ci*AP-1 pathway genes in response to MDP challenge. **(A)** Grass carp were injected with MDP in the presence or absence of the JNK inhibitor SP600125. Significant differences are indicated by * or # (*P* < 0.05). **(B)** Grass carp were challenged by PBS, MDP, MDP + carnosine, or MDP + Ala–Gln. The β-actin gene was used as a reference gene to normalize the expression level. Each bar represents the mean of the normalized expression levels of replicates (*N* = 3). Data without shared letters were significantly different (*P* < 0.05).

When challenged by bacterial MDP, *Ci*JNK mRNA in the intestine increased rapidly and significantly within 6 h of MDP treatment, peaked at 24 h (7.7-fold, *P* < 0.01), declined gradually after 48 h, and returned back to its original level at 72 h (Figure 6A). The expression of *Ci*AP-1 in the MDP challenge group was significantly upregulated at 12 h post injection (10.6-fold, *P* < 0.01) and 24 h post injection (3.4-fold, *P* < 0.01) and maintained at a relatively low level from 48 to 72 h post injection (Figure 6B). To determine whether *Ci*JNK is involved in the MDP-induced intestinal immune response, the expression levels of grass carp inflammatory cytokines (IL-6, IL-8, and TNF-α) were detected after stimulation with MDP and JNK inhibitor (SP600125) via qRT-PCR. As shown in Figure 7A, the MDP-induced expression levels of the intestinal IL-6, IL-8, and TNF-α genes were significantly decreased after the activity of the JNK pathway inhibited by SP600125. Moreover, the regulatory mechanism underlying the bacterial MDP-induced expression of *Ci*JNK/*Ci*AP-1 and inflammatory cytokines was investigated by injection experiments with the nutritional dipeptide carnosine or Ala–Gln. The results showed that the mRNA expression of all selected genes (including *Ci*JNK, *Ci*AP-1, IL-6, IL-8, and TNF-α) in the intestine was significantly increased by MDP stimulation (*P* < 0.05); however, the inductive effect of MDP could be significantly inhibited by carnosine or Ala–Gln treatment (Figure 7B). These data imply that the nutritional dipeptide carnosine or Ala–Gln may act as effective regulators to alleviate the bacterial MDP-mediated intestinal inflammatory response.

### *Ci*JNK Associates With *Ci*MKK4/*Ci*MKK7 Involved in the Activation of the AP-1 Signaling Pathway

**Figure 8:**
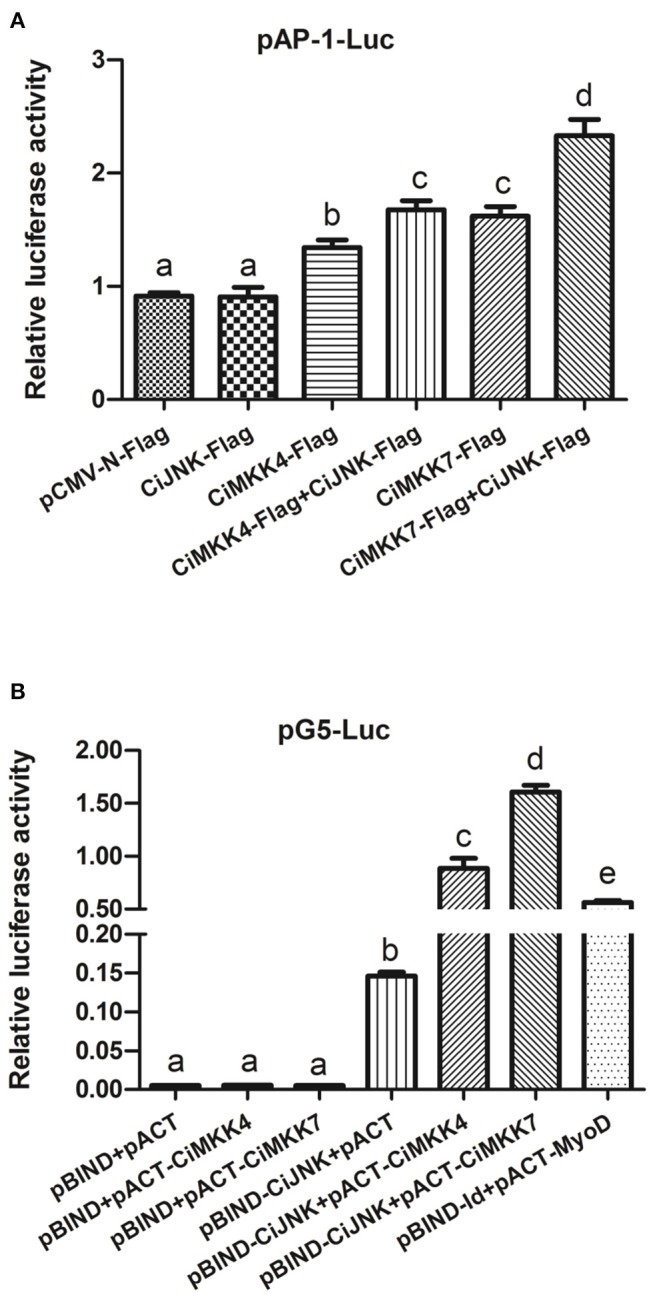
Overexpression analysis of *Ci*JNK and *Ci*MKK4/*Ci*MKK7 in HEK293T cells. HEK293T cells were transiently co-transfected with pRL-TK (10 ng per well), reporter vector (100 ng per well), and expression vector (250 ng per well). **(A)** The effects of *Ci*JNK and *Ci*MKK4/*Ci*MKK7 expression on the activity of the AP-1 reporter gene. **(B)** A direct protein–protein interaction between *Ci*JNK and *Ci*MKK4 or *Ci*MKK7 was found using a mammalian two-hybrid system. Significant differences are indicated by different letters (*P* < 0.05).

The luciferase reporter results showed that, compared with the empty vector pCMV-N-Flag (negative control), the transcriptional activation of AP-1-Luc in cells transfected with *Ci*MKK4-Flag or *Ci*MKK7-Flag alone was significantly enhanced (*P* < 0.05), while *Ci*JNK-Flag produced no obvious effect on the AP-1 signaling pathway in HEK293T cells (*P* > 0.05). When *Ci*JNK-Flag was co-transfected with *Ci*MKK4-Flag or *Ci*MKK7-Flag, the activation effects on the AP-1 signaling pathway were significantly higher compared with cells transfected with *Ci*MKK4-Flag or *Ci*MKK7-Flag alone (*P* < 0.05, Figure 8A), implying that *Ci*JNK may associate with *Ci*MKK4/*Ci*MKK7 in the activation of the AP-1 signaling pathway. The interaction between *Ci*JNK and *Ci*MKK4/*Ci*MKK7 was further investigated by conducting a mammalian two-hybrid assay in HEK293T cells. As shown in Figure 8B, it was found that the pACT-*Ci*MKK4 + pBIND-*Ci*JNK group, similar to the positive control pACT-MyoD + pBIND-Id group, also showed significantly upregulated pG5 luciferase reporter activity compared with the negative control pACT/pBIND, pBIND-*Ci*JNK + pACT, and pBIND + pACT-*Ci*MKK4 (*P* < 0.05). Similarly, the relative luciferase activity of the transfected pACT-*Ci*MKK7 + pBIND-*Ci*JNK cells was significantly higher than that of the pACT/pBIND, pBIND-*Ci*JNK + pACT, and pBIND + pACT-*Ci*MKK7 (*P* < 0.05). These results indicated that the *Ci*JNK protein could directly interact with the *Ci*MKK4 or *Ci*MKK7 protein in HEK293T cells.

**Figure 9:**
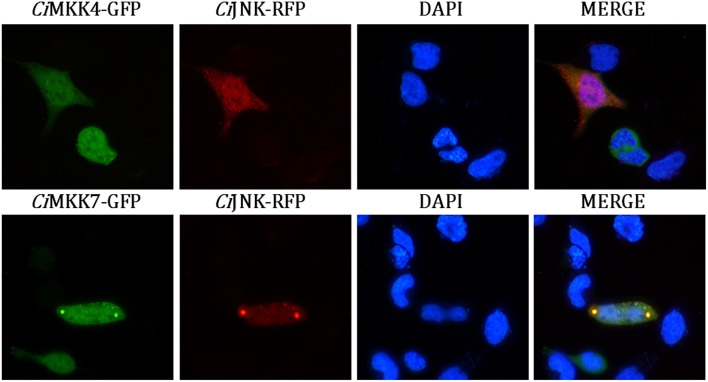
Subcellular co-localization analysis of *Ci*JNK and *Ci*MKK4/*Ci*MKK7 in HEK293T cells. The location of the *Ci*JNK-RFP protein is shown by the red fluorescence, and *Ci*MKK4-GFP/*Ci*MKK7-GFP proteins are indicated by the green fluorescence. The nucleus location is indicated by DAPI staining (blue).

Co-localization analysis of *Ci*JNK-RFP with *Ci*MKK4-GFP or *Ci*MKK7-GFP was performed to further verify the interaction between *Ci*JNK and *Ci*MKK4 or *Ci*MKK7 in HEK293T cells. As shown in Figure 9, a bright signal occurred with the co-transfection of *Ci*JNK and *Ci*MKK4 or *Ci*MKK7, indicating that *Ci*JNK could co-localize with the *Ci*MKK4/*Ci*MKK7 protein in HEK293T cells. Taken together, these findings suggest that *Ci*JNK is involved in the regulation of the AP-1 signaling pathway by interacting with specific upstream MKKs (*Ci*MKK4 and *Ci*MKK7).

## Discussion

Numerous studies have demonstrated that JNKs play an essential role in the immune defense process in response to pathogen immunological challenges in mammals (26, 27). However, data on the functions of fish JNKs in innate immunity, especially in intestinal inflammation, are still quite limited. In the present study, a fish JNK gene (*Ci*JNK) was first cloned and identified from the intestine of grass carp, suggesting that a conversed JNK signaling pathway might also exist in freshwater fish. A typical conserved TPY motif, which has been demonstrated to be responsible for the phosphorylation of JNK by upstream MKKs (MKK4 or MKK7) (34, 35), was observed in the activation loop of the presented *Ci*JNK protein. Previously, it has been shown that several duplication events occurred in the evolutionary process of JNK genes. The size of the known JNK family genes ranges from one to three copies, with one in shrimps (15), scallop (18), and oysters (16) and three (JNK1, JNK2, and JNK3) in mammals and fishes (28, 36). Multiple sequence alignment and phylogenetic analysis revealed that *Ci*JNK shares high sequence homology with the reported fish homologs and belongs to the JNK1 subfamily. Transcription factors such as NF-κB, AP-1, and STAT3 were shown to be involved in the regulation of the expression of numerous immune genes during pathogenic challenge. It is interesting that these transcription factor binding sites were observed in the 5′-flanking regions of the *Ci*JNK gene, suggesting that *Ci*JNK may be involved in the immune-related response in grass carp.

In mammals, it has been demonstrated that only the JNK1 and JNK2 genes of the JNK family are broadly expressed, and the JNK3 is more tissue specific (19). However, recent research has shown that fish JNK3 is also a ubiquitously expressed gene, and its transcripts have been detected in the brain, gill, skin, liver, and muscle (28). Broad expression patterns have also been observed in invertebrate JNKs, including *Lv*JNK from *Litopenaeus vannamei* (15) and *Cg*JNK from *C. gigas* (16). In our study, results showed that *Ci*JNK was expressed in all tested adult tissues, including the blood, intestine, spleen, heart, gill, kidney, muscle, and liver, which is consistent with previous reports on other animals. The universal distribution of *Ci*JNK may indicate that is has a broader, more generalized role in numerous physiological processes in grass carp. The fact that the highest expression level of *Ci*JNK was found in the liver implies that the liver may play a role in biological processes related to *Ci*JNK. In addition, *Ci*JNK was found to be expressed at a very low level in the spleen, intestine, and kidney, which are considered to be the primary immune tissues and play key roles in the host immune defense responses to pathogen challenge, indicating that *Ci*JNK may not be critical for physiological processes in these immune-related tissues under non-immune-challenged conditions. Previous studies have detected the expression of jnk1 mRNA and protein in zebrafish embryos at different developmental stages by RT-PCR and Western blot analysis (36). In a study with scallops, Sun et al. reported that *Py*JNK was broadly expressed in 10 different embryonic and larval stages in the Yesso scallop, *Patinopecten yessoensis* (18). These previous findings strongly suggest that the JNK pathway is involved in the development process in various species. The possible development-related functions of the JNK pathway in grass carp were investigated by measuring the mRNA expression profile of *Ci*JNK during different developmental stages by qRT-PCR. We found that *Ci*JNK transcripts were ubiquitously expressed at all developmental stages tested and showed a significant increase at the gastrula stage, indicating that *Ci*JNK might play a role in the embryonic development of grass carp.

MDP (*N*-acetylmuramyl-l-alanyl-d-isoglutamine) is a natural and minimal immunoreactive peptide released during peptidoglycan degradation, which is found in the cell wall of all Gram-positive and Gram-negative bacteria. Bacterial MDP has been shown to be transported by intestinal oligopeptide transporter 1 (PepT1) in epithelial cells, which can significantly induce inflammatory responses in animal intestines. Upon the PepT1-mediated transport in intestinal epithelial cells, MDP could be further recognized by the intracellular nucleotide-binding oligomerization domain 2 (NOD2) protein and then by activated receptor-interacting serine/threonine-protein kinase 2 (RIP2) to induce the production of inflammatory cytokines that participate in the intestinal immune response (37, 38). Over the past several years, much progress has been made in understanding the immune function of the PepT1-NOD2/RIP2 pathway in bacterial MDP-induced intestinal inflammation in vertebrates (39, 40). However, details of the downstream signaling pathway of PepT1-NOD2/RIP2 in MDP-induced intestinal inflammation remain unclear and need further exploration. Therefore, in this study, the possible role of *Ci*JNK/*Ci*AP-1 was analyzed after MDP challenge in the intestines of grass carp. Our results show that both *Ci*JNK and the downstream transcription factor *Ci*AP-1 exhibited a strong response to MDP challenge, and their expression levels were significantly upregulated in a time-dependent manner in the intestine of grass carp. In addition, grass carp were challenged by MDP in the presence or absence of a JNK inhibitor (SP600125) to further determine the role of the JNK pathway in MDP-induced intestinal inflammation. The results indicate that injection with MDP alone significantly upregulated the mRNA expression of intestinal inflammation cytokines (IL-6, IL-8, and TNF-α) compared with the PBS group; however, these MDP-induced effects were inhibited by the JNK inhibitor SP600125. These data strongly imply that the JNK pathway plays an important role in MDP-induced intestinal inflammation in grass carp. Previous studies in mammals have demonstrated that PepT1 can transport both bacterial MDP and nutritional dipeptide in intestinal epithelial cells (37, 41). On the basis of this previous research, the regulatory mechanism underlying MDP-induced intestinal inflammation was investigated after injection with the bacterial MDP and the nutritional dipeptide. The present results show that carnosine or Ala–Gln could significantly decrease the MDP-induced gene expression levels of the intestinal JNK pathway and inflammatory cytokines in grass carp, indicating that a nutritional dipeptide could effectively inhibit the PepT1-mediated epithelial transport of bacterial MDP. Previous studies have demonstrated that *A. hydrophila* is a Gram-negative bacterium that is recognized as one of the major pathogens threatening grass carp aquaculture (42). We found that challenge with *A. hydrophila* significantly upregulated the transcripts of *Ci*JNK and *Ci*AP-1 in the intestine, indicating the involvement of the *Ci*JNK/*Ci*AP-1 pathway in the bacteria-mediated intestinal response in grass carp. All these findings may provide some new insight into the prevention and treatment of bacteria-induced intestinal inflammatory diseases in aquatic animals.

Earlier studies have demonstrated that mammalian JNKs can be activated by the phosphorylation of Thr and Tyr residues through upstream dual-specificity MKK4 or MKK7 during immune challenges. Upon phosphorylation by MKKs, JNKs can then trigger the activation of the downstream transcription factor AP-1 and ultimately regulate the expression of downstream target genes (43, 44). It has been reported that JNK-induced AP-1 activation plays a key role in inflammation and the immune response (45, 46). However, whether the JNK/AP-1 pathway exists in low vertebrates, especially in bony fish, remains unclear. Our luciferase reporter assay showed that the overexpression of JNK alone could not activate the AP-1 pathway compared with the control in HEK293T cells. However, grass carp JNK could significantly enhance the MKK4- and MKK7-induced activity of the AP-1 reporter gene, implying that *Ci*JNK may associate with *Ci*MKK4/*Ci*MKK7 in the activation of the AP-1 signaling pathway. To unravel the activation mechanisms of *Ci*JNK and *Ci*MKK4/*Ci*MKK7 on the AP-1 pathway, we performed a mammalian two-hybrid assay in HEK293T cells. The results showed that there are very strong protein–protein interactions between *Ci*JNK and *Ci*MKK4 or *Ci*MKK7. The co-localization analysis further indicated that *Ci*JNK could co-localize with the *Ci*MKK4 or *Ci*MKK7 protein in HEK293T cells. In our previous studies, *Ci*MKK4 and *Ci*MKK7 were shown to be involved in the intestinal immune response to MDP challenge in grass carp. Taken together, these data suggest that *Ci*JNK may be involved in the regulation of the AP-1 signaling pathway by interacting with upstream *Ci*MKK4 and *Ci*MKK7 in MDP-induced intestinal inflammation.

In summary, the present work first reports the presence of a functional *Ci*JNK signaling pathway in grass carp, *C. idella*. Sequence analysis revealed that the *Ci*JNK protein contains a conserved dual-phosphorylation motif (TPY) in a serine/threonine protein kinase (S_TKc) domain. Quantitative RT-PCR showed that *Ci*JNK mRNA was broadly expressed in all tested tissues and embryonic developmental stages of the grass carp. The transcript levels of *Ci*JNK/*Ci*AP-1 were significantly upregulated by *A. hydrophila* and MDP challenge in the intestine. Furthermore, the JNK pathway was shown to be involved in the regulation of bacterial MDP-induced expression levels of inflammatory cytokines (IL-6, IL-8, and TNF-α) in the intestine. Moreover, the nutritional dipeptide carnosine and Ala–Gln could alleviate bacterial dipeptide MDP-induced intestinal inflammation in grass carp. Finally, subcellular localization and dual-reporter assays demonstrated that *Ci*JNK could associate with *Ci*MKK4 or *Ci*MKK7 in the regulation of the AP-1 signaling pathway. Collectively, these data indicate that the JNK/AP-1 pathway is involved in MDP-induced intestinal inflammation, which may provide new insight into the pathogenesis and prevention of inflammatory bowel disease.

## Data Availability Statement

The cDNA sequence of *Ci*JNK was submitted to the NCBI under GenBank accession number AYN79349.1.

## Ethics Statement

The animal study was reviewed and approved by the committee on animal care of Changsha University.

## Author Contributions

FQ and ZL designed the experiments and wrote the manuscript. WX, ZD, and YX conducted the experiments. JT and ZC analyzed the data. WL, DX, DZ, JF, and ZZ modified the manuscript. All authors reviewed and approved the final manuscript.

### Conflict of Interest

The authors declare that the research was conducted in the absence of any commercial or financial relationships that could be construed as a potential conflict of interest.
