# Supplementary material for: Fish c-Jun N-Terminal Kinase (JNK) Pathway Is Involved in Bacterial MDP-Induced Intestinal Inflammation
Source: Front Immunol. 2020 Mar 30;11:459. doi: 10.3389/fimmu.2020.00459 (PMC7134542; doi:10.3389/fimmu.2020.00459)
Supplement: Supplementary file 1 [file Data_Sheet_1.PDF]

Fig. S1.

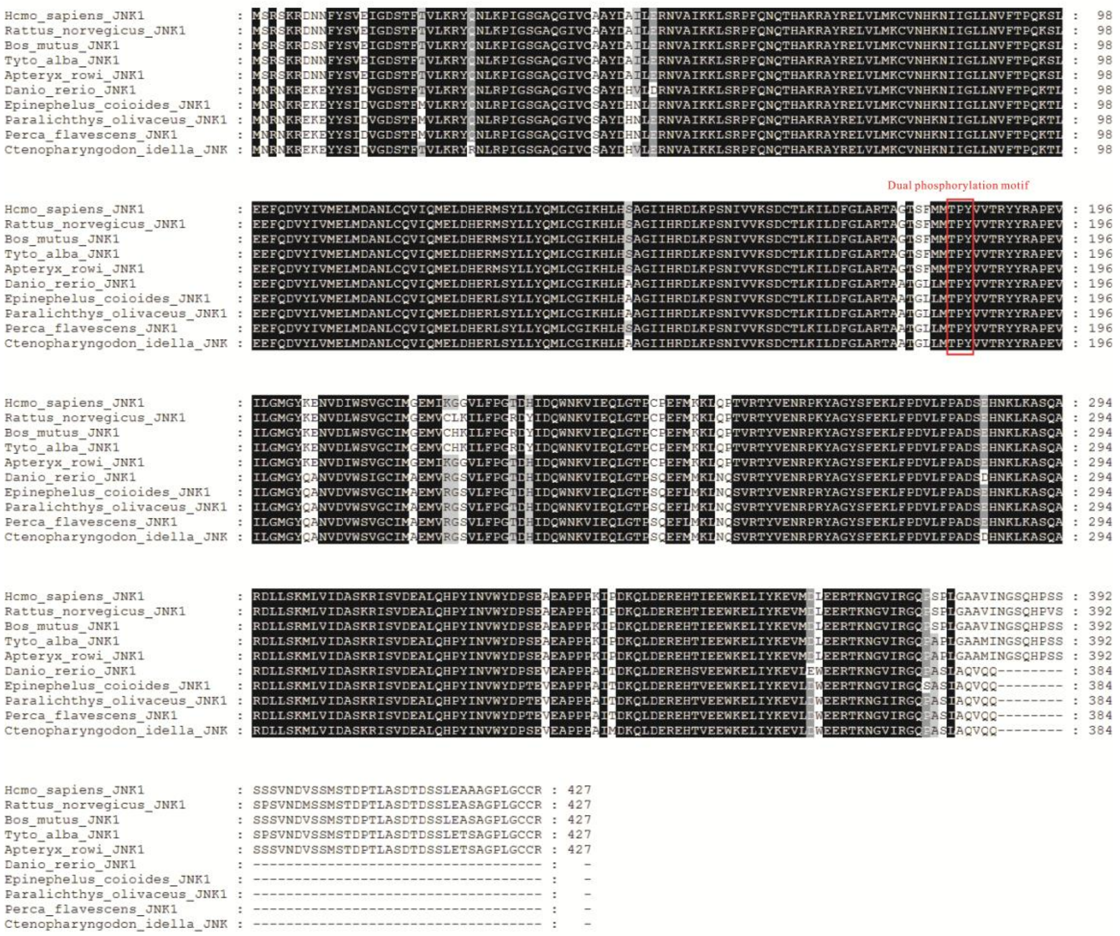

Fig. S1. Multiple alignment of the amino acid sequences of JNK from grass carp and other species. (A) Identical and similar residues are shaded in black and gray, respectively. The conserved TPY motif is shown by red boxes.
